# The Frequency of Double Expresser in Selected Cases of High Grade Diffuse Large B-Cell Lymphomas

**DOI:** 10.31557/APJCP.2020.21.4.1103

**Published:** 2020-04

**Authors:** Maria Naseem, Muhammad Asif, Muhammad Tahir Khadim, Hafeez Ud-Din, Shahid Jamal, Iman Shoaib

**Affiliations:** 1 *Department of Histopathology, *; 2 *Department of Forensic Medical Sciences, Molecular Pathology, Armed Forces Institute of Pathology, Rawalpindi, Pakistan. *

**Keywords:** Immunohistochemistry (IHC), Diffuse Large B Cell Lymphoma (DLBCL), Double expresser (DEL), R-CHOP

## Abstract

**Background:**

Diffuse large B-cell lymphomas (DLBCL) are fast-growing non-Hodgkin lymphomas that affect B-lymphocytes. Double expressor DLBCL is the concomitant expression of Myc and Bcl-2 proteins during lymphomas which results in poor prognosis of patients. This study aimed to determine the frequency of double expresser in high grade diffuse large B-cell lymphomas.

**Materials and Methods:**

The study was conducted on 74 cases (54 males (68.4%) and 20 females (25.3%)) of DLBCL between August 2018 to January 2019. The mean age of the 74 patients was 51.7 years + 18.5. Expression of proteins c-Myc, Bcl-2 and Bcl-6 were evaluated by immunohistochemistry. The involvement of primary lymph node was reported in 38 cases (51.3%) whereas, extra nodal site was observed in 22 cases (29.7%). Among the primary sites, the cervical lymph node enlargement was the most frequent site of presentation.

**Results:**

The rearrangement pattern was studied among 74 patients, 35 (47%) were found to have either one of the rearrangements i.e. Myc, Bcl-2, or Bcl-6. On the other hand, 14 (18.9%) had shown double rearrangements i.e. Bcl-2 and c-Myc (11 cases) and Bcl-6 and c-Myc (3 cases). The Bcl-2 and Bcl-6 rearrangements were demonstrated by 12 cases whereas 2 cases (2.7%) indicated all three types of rearrangements i.e. c-Myc, Bcl-2, and Bcl-6. In 11 cases the Bcl-2 and c-Myc rearrangements were found to be Bcl-2 > 50% and c-Myc > 40% and demonstrating the overall frequency of double expressers as 14.8%. The prognosis of the mentioned cases was extremely poor, median survival of 10 months. Conclusion: The concurrent expression of Bcl-2 and c-Myc was found to be 14% (level of expression for Bcl-2 > 50% and c-Myc > 40%) which is potentially a significant health burden and an emerging threat.

## Introduction

Lymphoma is a type of cancer that involves cells of the immune system, called lymphocytes which grow and multiply in an uncontrolled manner (Chiu and Blair, 2009). Several factors have been considered responsible in the increased incidence of lymphoma such as; advancement in diagnostic procedures, congenital and acquired immunodeficiency state, viral infections (Human Immunodeficiency Virus (HIV), Epstein Barr Virus (EBV), Hepatitis C Virus (HCV), Human T Leukemia Virus (HTLV), exposure to certain chemicals including pesticides, hair coloring agents, and herbicides e.g 2,4-dichlorophenoxyacetic acid (2,4-D) (Chiu and Blair, 2009). There are two main categories of lymphomas: Hodgkin (HL) and Non-Hodgkin lymphoma (NHL). Hodgkin lymphoma has been further divided into five subtypes on the basis of types of cells and their morphological arrangements (Alizadeh et al., 2000). Non-Hodgkin lymphomas are of B and T-cell types and further fall into categories on the basis of immunohistochemical staining (Alizadeh et al., 2000). 

The B-cells are derived from the hematopoietic stem cells in the bone marrow and acquire cell surface markers including Clusters of differentiation (CD) proteins that can be used in the identification of various maturation stages (Young et al., 1999). However, gene rearrangement is considered as the major cause affecting the expression of specific surface markers on B and T-cells. Gene rearrangement is a type of chromosomal abnormality that involves a structural change in the native chromosome. After gene rearrangements for immunoglobulins and expression of specific surface markers, the mature naïve B-cells leave the bone marrow and settles into the lymphoid tissue (Young et al., 1999). c-Myc is known to be a “master regulator” of the genes that are involved in metabolism and cell cycle growth initiating and maintaining molecular mechanisms (Rezzoug et al., 2016). It is a transcription factor located on chromosome 8q24 that is crucial for early B-cell development in the bone marrow by activating a transcription factor required for the maintenance of B-cells identity (Nguyen et al., 2017). The chromosomal translocation is a type of abnormality in which chromosome breaks and its one portion gets reattached to another chromosome. In B-cell lymphomas, chromosomal translocation results in deregulation of Myc and Bcl-2 resulting in inhibition of apoptosis (Nguyen et al., 2017). 

Diffuse Large B-cell Lymphoma (DLBCL) constitutes a great majority of all Non-Hodgkin lymphomas (NHL). In the west (Germany, Canada, Australia), it constitutes 30-40% of all B-cell lymphomas (Abid et al., 2005). According to global cancer facts and figures, the estimated age-standardized incidence and mortality rates (per 100,000), 2008, for NHL was 10.3 and 3.6 respectively for developed countries (including, Australia, Northern America and Western and Northern Europe) and 4.2 and 3 for developing countries (Asia and Eastern Europe) whereas for HL it is 2.2 and 0.4 in developed countries and, 0.9 and 0.6 in developing countries (Garcia et al., 2007). In the United States, NHL represents approximately 3.3% of all cancer deaths being the 7th leading cause of cancer death (Garcia et al., 2007). The average incidence of NHL is approximately 0.5% per year and highest among people aged 65-74 years (Garcia et al., 2007). In Pakistan, according to Karachi cancer registry in year 2002, the annual incidence of NHL is 8.4/100,000 in males and 6.5/100,000 in females (Pervez, 2012). 

Double-expresser lymphoma (DEL), also known as double-positive lymphoma is defined based on immunohistochemical stains. On more than a specified proportion of tumor cells with Myc and Bcl-2 staining, both are overexpressed and have dismal prognosis (Scott et al., 2015). 

In the clinical diagnosis and prognosis of aggressive B-cell lymphomas, the detection of c-Myc gene translocation and subsequent protein expression has become indispensable. There are two techniques that are currently available for the detection of c-Myc gene abnormalities; Conventional karyotyping and Fluorescence in situ hybridization (FISH). Karyotyping is used to assess ploidy, deletions, and translocations. Whereas, Fluorescence in situ hybridization (FISH) can detect Myc translocations but it fails to detect its deregulation due to other mechanisms (Swerdlow, 2014). Immunohistochemistry has been validated to predict Myc rearrangements for use formalin-fixed paraffin-embedded (FFPE) tissue, targeting the N-terminus of the Myc protein by a monoclonal antibody (Tapia et al., 2011). However, DLBCL is like a two-edged sword, being an aggressive tumor if untreated or treated with rituximab in combination with cyclophosphamide, doxorubicin, vincristine and prednisolone chemotherapy (R-CHOP), it is rapidly fatal and has increased risk of central nervous system (CNS) involvement. None the less, with intensive, combination chemotherapy, it is potentially curable (Savage et al., 2009). Identification of dual expresser status is helpful in a high-risk group that needs CNS-directed evaluation and prophylactic strategies (Sarwar and Saqib, 2017). 

The aim of the current study was to identify the presence of co-expression of proteins involved in c-Myc with Bcl-2. The required criteria of expression of c-Myc with Bcl-2 (which was >40%) of cells stains positive on IHC was investigated to classify as double expresser lymphoma (DEL). 

## Materials and Methods

*Population and Sample size*

This study was conducted on previously diagnosed patients with high-grade DLBCL to observe the frequency of cases overexpressing both the Bcl-2 and c-Myc (>40% of cells stains positive on IHC).The WHO calculator was used for calculating the sample size. The estimated sample size calculated was 74 cases considering; given prevalence of 4%, level of confidence 95%, margin of error 5%, at required precision of 6%. The calculations were based on the given frequency of Non-Hodgkin lymphomas (NHL) in a study conducted by Muhammad Rehan Sarwar et al (Sarwar et al., 2017).

*Location and type of study*

Armed Forces Institute of Pathology (AFIP) is a major referral center of Pakistan, receiving a huge proportion of specimens from the Northern Province including Rawalpindi. This study was cross-sectional conducted at department of Histopathology, Armed Forces Institute of Pathology (AFIP) Rawalpindi, Pakistan from August 2018 to January 2019. 

*Inclusion and exclusion criteria*

The 74 cases were selected from the previously diagnosed cases of high-grade B-cell lymphoma having requisite data and complete workup (including LCA, CD20, CD3, CD5, CD10, CD15, CD30, CD23, CD43, Cyclin D1, PAX8, Bcl-6 and Ki-67). The cases diagnosed as low-grade lymphoma, Burkitt’s lymphoma and with incomplete or without immunohistochemical workup were excluded. 

*Variables*

During sample collection and processing, three essential variables were considered as part of study including; age, gender and site of involvement. No statistical evaluation was further conducted using the variables to establish a statistical relationship. Ethical approval for this study was obtained from the institutional review board of the Armed Forces Institute of Pathology (AFIP) Rawalpindi.

*Procedure*

In this study, the additional panel of c-Myc, Bcl-2, and Bcl-6 with positive and negative controls were applied by Dako Envision method on large tumors with the well-preserved area. Slides were interpreted by qualified Histopathologist and the statistical data was calculated including patients mean age, frequency and percentage of Bcl-2 and c-Myc rearrangements.

## Results

*Variables*

**Figure 1:**
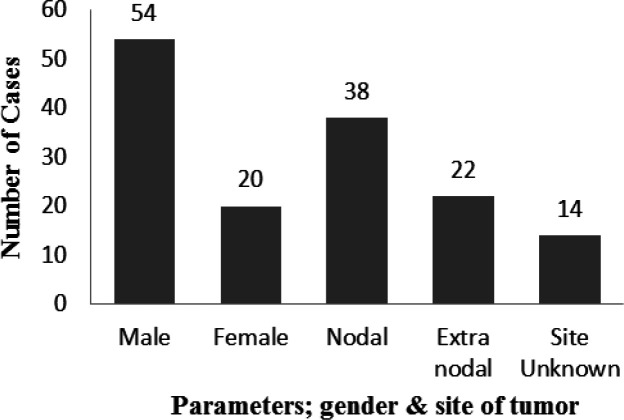
The Graphical Representation of Parameters Including Gender and Site of Tumor among 74 Cases (Mean Age 51.7 year + 18.5)

In this study, 74 patients with DLBCL were studied, out of which included; male 54 (68.4%) and female 20 (25.3%) with mean age average of 51.7 years + 18.5. Three parameters were considered including; age, gender and the involvement of primary lymph node and extranodal lymphoma. No further investigation was conducted in this current study to find any significant association involved with the age, gender, and site of involvement to the overall survival. Referring to Figure 1 is the representation of the parameters; gender and site of tumor location against respective number of cases. 

*Involvement of site*

As shown in Figure1, The primary lymph node involvement was reported in 38 (51.3%) and extranodal site was observed in 22 (29.7%) cases. Among the primary sites, the cervical lymph node enlargement was the most frequent site of presentation. Out of 74 patients, in 14 (18.9%) cases site of involvement was not known.

*Rearrangement pattern of proteins*

The rearrangement patterns of proteins; Myc, Bcl-2 and Bcl6 were studied among 74 patients, as shown in Table 1. The 29 (39.1%) cases were found to have either one of the rearrangements i.e. Myc, Bcl-2, or Bcl-6. On the other hand, 14 (18.9%) had shown double rearrangements i.e. Bcl-2 and c-Myc (11 cases) and Bcl-6 and c-Myc (3 cases). The rearrangement of Bcl-2 and Bcl-6 were seen in 12 cases whereas 2 cases (2.7%) indicated all three types of rearrangements i.e. c-Myc, Bcl-2, and Bcl-6. No expression of rearrangement was observed among 17 cases. 

*Expression of rearrangement of proteins*

The level of expression of the rearrangements was determined by immunohistochemistry staining. Referring to table 2.0, the tabulated results indicate the level of expression varying among different cases. In 11 cases with Bcl-2 and c-Myc rearrangements were found to be Bcl-2 > 50% and c-Myc > 40%. These were classified as double expresser lymphomas (DEL) and were included in the analysis by demonstrating the overall frequency of double expressers as 14.8%. 

*Prognosis and treatment*

**Figure 2:**
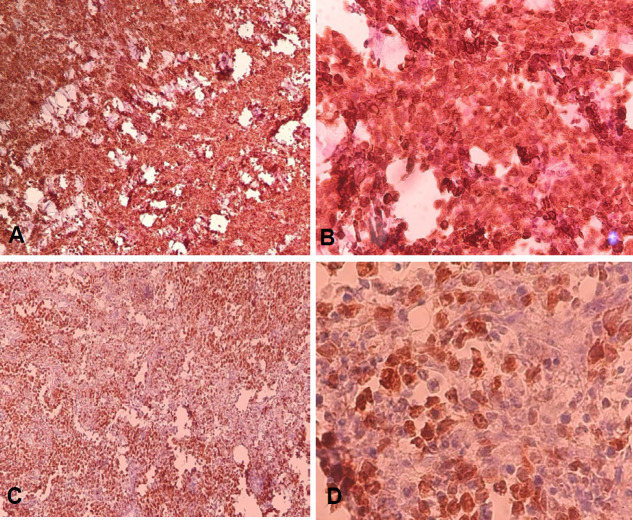
Images A and B Represent the Nuclear Pattern of Strong Intensity Staining of Bcl-2 in more than 50% of Cells (A= 100X magnification, B= 400X magnification).

The prognosis of the mentioned cases was extremely poor, median survival of 10 months. The rearrangement c-Myc and Bcl-2 reportedly showed association with significantly worse overall survival (P = 0.01) and are refractory to conventional chemotherapy and stem cell transplant. These rearrangements also predicted significantly shorter overall survival (P = 0.04) (Akyurek et al., 2012). The concurrent expression of Bcl-2 and c-Myc under magnification of 100X and 400X is represented in Figure 2. 

**Table 1:** The Pattern of Rearrangements Observed in Cases

Patterns	Number of Cases
Bcl-2 / Bcl-6 / c-Myc	29
Bcl-2 and c-Myc	11
Bcl-6 and c-Myc	3
Bcl-2 and Bcl-6	12
Bcl-2 + Bcl-6+ c-Myc	2
No rearrangements	17

**Table 2:** Expression of Rearrangements Observed in Cases under Investigation

Number of cases	Expression of Rearrangements (% age)
11	c-Myc> 40
	Bcl-2 > 50

Image C and D representthe nuclear pattern of strong intensity staining of c-Myc in more than 40% of cells (C= 100X magnification, D= 400X magnification).

**Table 3:** Study Comparisons for Cutoff Value, Expression and Concurrent Expression of Bcl-2 and c-Myc

Author	Year	Cutoff value (% age)	Expression of Bcl-2 & c-Myc (% age)	Concurrent expression of Bcl-2 & c-Myc (% age)
Barranset al.,	2010	-	14	-
			Total number of cases 245	
Green et al.,	2012	0-70	-	-
		Bcl-2		
				
Johnson et al.,	2012	-	44 Bcl-2	21 Bcl-2
			29 Myc	2q Myc
			Total number of cases 167	
Valera et al.,	2013	40 Myc IHC positivity	-	4 Bcl-2
				4 Myc
				
AFIP	2019	c-Myc ≥40	5 Bcl-2	14 Bcl-2
		Bcl-2 ≥50	14 Myc	14 Myc
			Total number of cases 74	

## Discussion

In this study, we observed the frequency of double expresser lymphoma (DEL) among high-grade DLBCL cases. The three major parameters including age, gender, and site of involvement were considered. The mean age of the 74 patients was calculated by SPSS online software and found to be 51.7 years + 18.5 (referring to Table 1). The current study was conducted to aim for expression (acquired criteria >40% of cells stains positive on IHC) and co-expression of Myc with Bcl-2. Referring to result section in Tables 1 and 2, the expression of Bcl-2, Bcl-6, and c-Myc in 74 patients were diagnosed with cases of DLBCL. Valera et al., (2013) has used the cutoff value of 40% in one of the studies for Myc IHC positivity and Green et al., (2012) included the cutoff range for Bcl-2 ranging from 0-70%. In the current study, we used the cutoff value of c-Myc > 40% and Bcl-2 > 50% (referring to Table 2). Johnson et al., (2012) reported in a study that used combination of Bcl-2 and Myc in 167 patients of DLBCL followed by validation of study in another group of 140 patients. Bcl-2 and Myc proteins were detected in 44% and 29% out of 167 patients, respectively and co-expression of Bcl-2 and Myc was found in 21% of cases. On the other hand, our study showed Bcl-2 and Myc proteins weredetected in 5% and 14%, respectively. These results are comparable to the study conducted by Barrans et al., (2010), in which Myc protein was detected in 14% cases out of 245 and it was found to be a strong adverse prognostic factor in patients treated with standard RCHOP chemotherapy. Concurrent expression of Bcl-2 and c-Myc (referring to Figure 2) in current study was 14% which is comparatively lower than the results of another study by Johnson et al., (2012). Valera et al., (2013) have reported, out of 219 cases involved in DLBCL the concurrent expression of Bcl-2 and Myc was found to be in only 4% cases. Although, this was quite lower value than our results they have concluded that the tumors with concurrent expression of both Bcl-2 and Myc had the worst prognosis as compared to double negative tumors. Comparable to this study, Au et al., (1999) in their findings with several small case series have described the very poor overall survival (OS) in patients with Bcl-2+/Myc+ lymphomas.

DLBCL with overexpression of Myc and Bcl-2 represents a unique category of high-grade lymphoma with inferior overall survival. As these lymphomas are refractory to chemotherapy and immunotherapy, risk of CNS involvement and relapse rates are very high. Early detection, prompt treatment, and CNS prophylaxis are the mainstay of the management of this unique category of DLBCL. As referring to table 3. an overview of study comparison between prior results mentioned above and with the current study findings is represented. 

In conclusion, the findings from our study conclude the concurrent expression of Bcl-2 and c-Mycwas found to be 14% (level of expression for Bcl-2 > 50% and c-Myc > 40%) that ispotentially a significant health burden and an emerging threat. Although parameters may directly or indirectly be involved in gene regulation and chromosomal translocation yet, no further investigation was conducted in this current study to find any significant association involvedwith the age, gender, and site of involvement tothe overall survival. With the expectations to have increase in the prevalence of DEL due to adaptations of such lifestyle factors, prophylactic measures must be considered. However, as part of the mandatory initial IHC panel of DLBCL, c-Myc expression should be included. Early diagnosis and effective treatment compliment theoverall rate of curing time. According to our study combination chemotherapy and CNS prophylaxis should be part of the treatment guidelines.
